# Effect of armed conflict on vaccination: evidence from the Boko haram insurgency in northeastern Nigeria

**DOI:** 10.1186/s13031-019-0235-8

**Published:** 2019-10-29

**Authors:** Ryoko Sato

**Affiliations:** Global Health and Population, Harvard T. H Chan School of Public Health, 90 Smith Street, 332-1, Boston, MA 02120 USA

**Keywords:** Conflict, Event study, Vaccination, Boko haram

## Abstract

**Background:**

Armed conflicts can have severe adverse effects on population health, both directly and indirectly, through the destruction of health care systems. This paper evaluates the causal effect of the Boko Haram insurgency in northeastern Nigeria on the vaccination rate.

**Methods:**

By matching children’s birth months and the months of armed conflict, we evaluate the effect of armed conflict on the vaccination rate of children. We use two datasets: the Nigeria Demographic and Health Survey (DHS) for vaccinations and the Uppsala Conflict Data Program (UCDP) Georeferenced Event Dataset (UCDP GED) for armed-conflict events.

**Results:**

We find a large negative effect of conflict events on the likelihood of vaccination; if an armed conflict occurs within 10 km from where a child resides, the odds that child receives any vaccination are 47.2% lower. Odds ratio for BCG and DPT1 is 0.55 and 0.52 respectively. We also find that armed conflicts have more impact among the non-educated population than among the educated.

**Conclusion:**

Overall, armed conflicts had a devastating effect on the vaccination of young children who were born at the time of these conflicts in northeastern Nigeria. A reduced vaccination rate puts the vulnerable population in affected areas at risk of contracting vaccine-preventable diseases.

## Background

Armed conflicts have severe adverse effects on population health [1]. The impact of armed conflicts can be direct, such as mortality and morbidity due to attacks. Armed conflicts can also indirectly affect population health, for example through the destruction of health care systems [2]. This indirect effect of armed conflicts can persist even after the conflicts are over because it takes time for health systems to recover.

Nigeria has struggled with constant conflicts among its highly diverse population in terms of religion, ethnicity, and political views [3]. One of the most notable and devastating conflicts in Nigeria in recent years is the Boko Haram insurgency. Boko Haram was created around 2002 in Borno state in northeastern Nigeria as a social movement to protest poverty [4]. Currently, Boko Haram is known as one of the most extreme Islamist groups in sub-Saharan Africa. Since beginning in 2009, the Boko Haram insurgency has killed more than 20,000 people. More than 2 million people have been displaced by the conflict [5].

Conflicts expose the population to the risk of contracting diseases [6]. Vaccination is the most effective way to prevent the outbreak of many diseases. However, it might be challenging to reach populations in conflict-affected areas to provide vaccination services. Because of the limited access to vaccination services during armed conflicts, such conflicts can have devastating effects on disease outbreaks. Despite the potential importance of conflicts, there is an extremely limited number of studies on the effects of conflicts on immunization [7–9]. Grundy and Biggs [8] used national and sub-national levels of vaccination coverage among 16 conflict-affected countries and qualitatively observed the consequence of conflict on immunization coverage. Mashal et al. [7] categorized geographical regions in Afghanistan based on insecurity and examined the association of insecurity with immunization coverage.

The existing literature lacks high-quality studies on the causal relationship between conflict events and the vaccination rate. Furthermore, the consequences of the Boko Haram insurgency on the population have rarely been studied [3, 10–13]. This study is the first to evaluate the effect of armed conflicts due to the Boko Haram insurgency on vaccination in northeastern Nigeria. This study uses detailed information about children’s birth months and the months that armed conflicts occurred.

## Methods

### Data

We use two data sets for the analysis. The first is the Nigeria Demographic and Health Survey (DHS) conducted in 2013 (National Population Commission and ICF International, 2014). The DHS contains information on the immunization status of respondents’ children who were under 5 years old at the time of the survey. The DHS also contains various sociodemographic characteristics such as age, educational attainment, marital status of mothers, and wealth level of households, as well as GPS coordinates for clusters where respondents reside. In this paper, the analysis focuses only on respondents and children in 3 states in Nigeria, Adamawa state, Borno state, and Yobe state, where the effects of the Boko Haram insurgency are considered the most devastating. The federal government of Nigeria declared a state of emergency in these 3 states at the peak of the Boko Haram insurgency in 2013.

The second dataset is the Uppsala Conflict Data Program (UCDP) Georeferenced Event Dataset (UCDP GED), which has information on armed conflict events that occurred between 1989 and 2017 all over the world [14]. UCDP GED has information on the timing of conflicts, GPS coordinates of conflict locations, and estimates of total fatalities resulting from each conflict event. For the analysis, we restrict the sample to events that occurred in Nigeria between 2007 and 2013 to match the timing of children’s vaccinations recorded in the DHS.

### Outcome variables

We use three indicators to capture the vaccination status: 1) take-up of at least one vaccine, 2) take-up of BCG, and 3) take-up of the first dose of DPT (DPT1). These are all dummy variables, which take a value of one if a respondent’s child receives the vaccine, based on self-reported records. Although the DHS data have information on the date of vaccination for each type of vaccine for each child, we do not use this information because the dates are missing for many children. The recommended immunization schedule for BCG is at birth and for DPT1 is 6 weeks after birth. We focus on BCG and DPT1 to evaluate the immediate effect of conflict events around the time of childbirth on vaccination.

### Main independent variable

The main independent variable is the occurrence of the conflict event. The conflict event is a dummy variable that indicates that at least one armed-conflict event occurred around the time of childbirth of a respondent within a radius of 10 km from the cluster where the respondent resides. We match the birth months of children and the months that armed conflicts took place. We arbitrarily define a cutoff radius of 10 km. As a robustness check, we also evaluate the effects of conflict within 15 km, 30 km, and 50 km. Results are consistent, regardless of the various cutoffs we use.

### Statistical analysis

We estimate the effect of armed conflicts at the time of childbirth on vaccination using the logistic regression in the following regression framework:

1 $$ {y}_{ij t}=\alpha +{\beta}_1{Conflict}_{ij t}+X^{\prime}\mu +{v}_j+{\varepsilon}_{ij} $$

where *y*_*ij*_ is the vaccination of a respondent’s child *i* who was born at time *t* in cluster *j*; *Conflict*_*ijt*_ indicates whether the armed conflict occurred in the month when the child of respondent *i* was born (*t*), within 10 km from the cluster of respondent *i* in cluster *j*. *X* includes various variables to control for the sociodemographic characteristics of the women, households, and children, such as age, education level, marital status, religion, number of household members, number of children under 5 years old, wealth level, place of residence (rural vs. urban), and the child’s age, gender, and birth order. We also control for the year and month of childbirth. *ε*_*ij*_ is an error term.

In addition to the standard model (1), we also evaluate the effects of conflict events that occurred some months after childbirth on vaccination using the following regression framework:

2 $$ {y}_{ij t}=\alpha +{\beta}_1{Conflict}_{ij\left(t+j\right)}+X^{\prime}\mu +{v}_j+{\varepsilon}_{ij} $$

where *j* =0, 1, 2, or 3. When *j* =0, the event occurred in the same month as the childbirth. In this case, Eq. (2) is identical to Eq. (1). When *j* =1, 2, or 3, the event occurred 1, 2, or 3 months after the childbirth, respectively. Because the recommended timing of DPT1 is 6 weeks after birth, we evaluate the effect of conflicts that took place after the child was born on vaccine take-up.

## Results

**Fig. 1:**
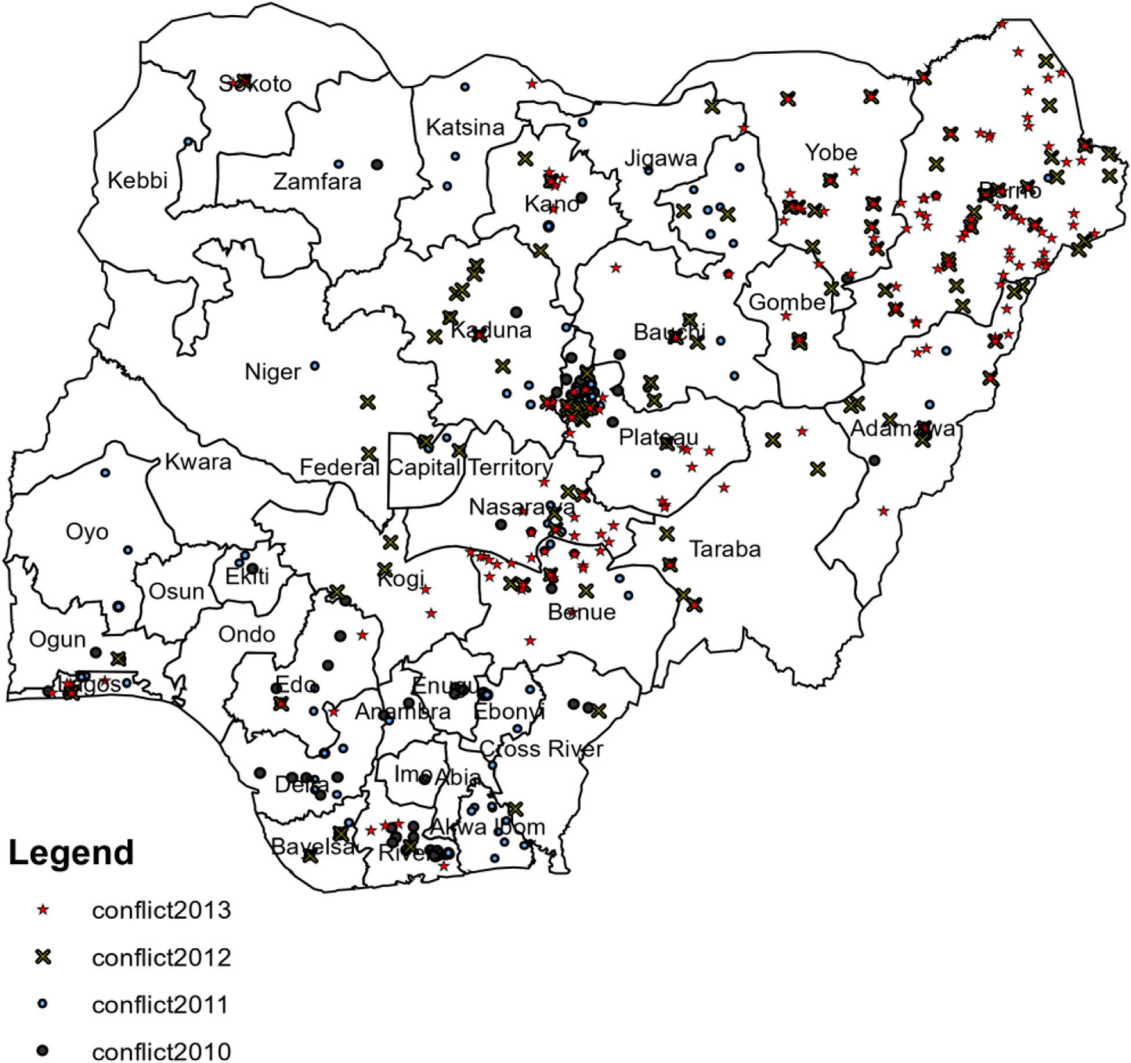
Conflict events by year. Each dot presents the armed-conflict event

Two thousand four hundred thirteen children, aged 0 to 59 months old, were born between 2007 and 2013 in the three northeastern states of Adamawa, Borno, and Yobe. Figure 1 geographically presents the incidence of conflict events by year. There is a high concentration of conflict events in the northeastern region of Nigeria, especially in 2012 and 2013.

Table 1 presents the prevalence of armed conflicts in the three northeastern states that occurred within 10 km from the clusters where respondents resided, according to the sociodemographic characteristics. Overall, 3.8% of children experienced conflict events within 10 km of their residence at the month of their birth. There is large variation in the occurrences of conflict events according to respondents’ characteristics. As the educational attainment of mothers rises, so does the prevalence of conflict. Among mothers with no educational attainment, the prevalence of conflict events is 2.7%, while 15.3% of mothers with education higher than secondary school experienced conflict events. Similarly, the prevalence of conflict events is high for the richest mothers, 19.3%, while among the poorer mothers the prevalence is less than 1 %. The Boko Haram insurgency mostly affects urban cities rather than rural areas; the conflict prevalence is less than 1 % in rural areas, while it is as high as 12% in urban areas.

**Table 1:** Prevalence of conflict

	Total # mothers (1)	Conflicts within 10 km at child delivery Proportions of mothers affected by conflicts (2)	Std. Dev (3)
Mother’s characteristics			
Education level			
No education	2297	0.027	0.163
Primary	453	0.035	0.185
Secondary	461	0.061	0.239
Higher	118	0.153	0.361
Wealth level			
Poorest	1187	0.002	0.041
Poorer	931	0.006	0.08
Middle	549	0.038	0.192
Richer	418	0.117	0.322
Richest	244	0.193	0.395
Residence			
Urban	974	0.122	0.328
Rural	2355	0.003	0.05

Notes: The sample is women from Borno, Yobe, and Adamawa state. “Conflict” takes 1 if the conflict occurred within 10 km from each mother

Appendix 1 presents the descriptive statistics of the sample. The mothers’ average age is 28.7 years old and 67.9% of mothers have no educational attainment, while only 3.8% of them have educational attainment higher than secondary school. Most mothers are Muslim (88.2%), while the rest are Christian. More than 95% (96.1%) of mothers are married. The average number of household members is 7.8 and the average number of children under five in a household is 2.4. Many of the sampled households are poor; most households belong to the poorest (34.9%) and poorer (26.9%) categories, while fewer are in the richest category (7.7%). The average age of children is 29.2 months and the average birth order of the children in the sample is 4.2. Slightly less than half of the children in the sample are girls (47.7%). On average, 49.1% of children have received a vaccination: 40.2% of them received BCG and 38.3% received DPT1.

**Fig. 2:**
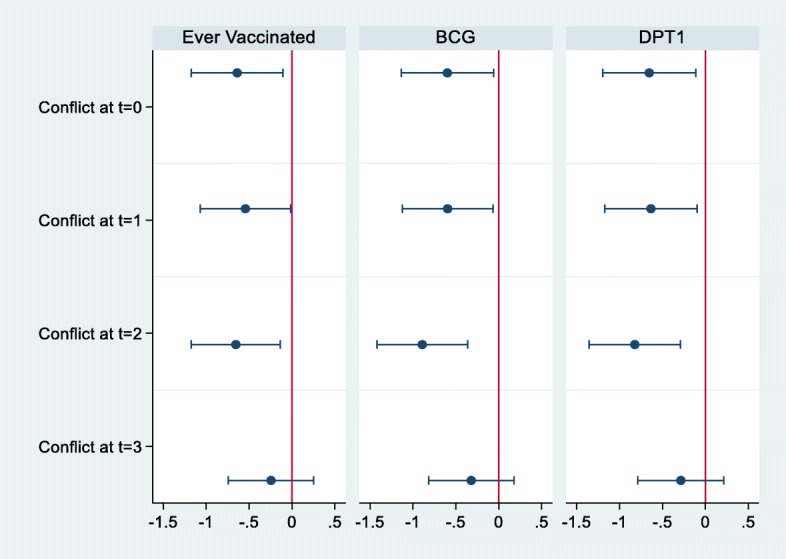
Effect of Conflict on Vaccination. The sample is 2413 mothers who are in Borno, Yobe and Adamawa state. The figure shows the estimated coefficients, and the estimate is based on the logistic regression. The independent variable “conflict” takes 1 if the conflict occurred within 10 km from each mother. t = 0 means conflict at Birth month. t = 1 means conflict at 1 month after birth. t = 2 means conflict at 2 months after birth. t = 3 means conflict at 3 months after birth. Covariates include child’s age, birth year, sex of child, birth order, age of mother, education level of mother, marital status, religion, number of HH members, number of children under 5, wealth level

Figure 2 and Table 2 present the main result for the effect of conflict events on vaccine take-up. Overall, the effect of conflicts around the time of childbirth on vaccination is significant and negative (Fig. 2). If a conflict event occurred in the same month as the childbirth, the odds of vaccination are 47.2% lower for any vaccination (Table 2 column 1), 45.0% lower for BCG (column 2), and 48.0% lower for DPT1 (column 3). If a conflict event occurred 1 month after the childbirth, the odds of vaccination are 41.9% lower for any vaccination (column 4), 45.8% lower for BCG (column 5), and 47.0% lower for DPT1 (column 6). If a conflict event occurred 2 months after the childbirth, the odds of vaccination are 48.0% lower (column 7) for any vaccination, 59.0% lower for BCG (column 8), and 56.1% lower for DPT1 (column 9). If a conflict event occurred 3 months after the childbirth, the effect of conflict events is not significant on the likelihood of children’s vaccination, regardless of the type of vaccine (column 10–12). Appendix 2 presents the full results, including all the covariates except for the children’s ages and birthdates. We find that education and wealth are especially strong predictors of children’s vaccination. Appendix 3 presents the main results, using 30 km as a cutoff distance for experiencing armed-conflict events instead of 10 km. The results are similar to those shown in Table 2.

**Table 2:** Effect of conflict on vaccination (odds ratio)

	Ever vaccinated (1)	BCG (2)	DPT1 (3)	Ever vaccinated (4)	BCG (5)	DPT1 (6)	Ever vaccinated (7)	BCG (8)	DPT1 (9)	Ever vaccinated (10)	BCG (11)	DPT1 (12)
Conflict at Birth month	0.528** [0.309,0.901]	0.550** [0.321,0.942]	0.520**[0.303,0.892]									
Conflict at 1 month after birth				0.581**[0.343,0.984]	0.552** [0.325,0.936]	0.530** [0.310,0.907]						
Conflict at 2 month after birth							0.520**[0.309,0.874]	0.410*** [0.242,0.696]	0.439*** [0.258,0.749]			
Conflict at 3 month after birth										0.783 [0.476,1.289]	0.726 [0.442,1.194]	0.751 [0.455,1.238]
N	2410	2405	2401	2410	2405	2401	2410	2405	2401	2410	2405	2401
Covariates	X	X	X	X	X	X	X	X	X	X	X	X

Notes: The sample is 2413 mothers who are in Borno, Yobe and Adamawa state. We use logistic regression. The independent variable “conflict” takes 1 if the conflict occurred within 10 km from each mother

Covariates include child’s age, birth year, sex of child, birth order, age of mother, education level of mother, marital status, religion, number of HH members, number of children under 5, wealth level

** significant at 5%, and *** significant at 1%

**Fig. 3:**
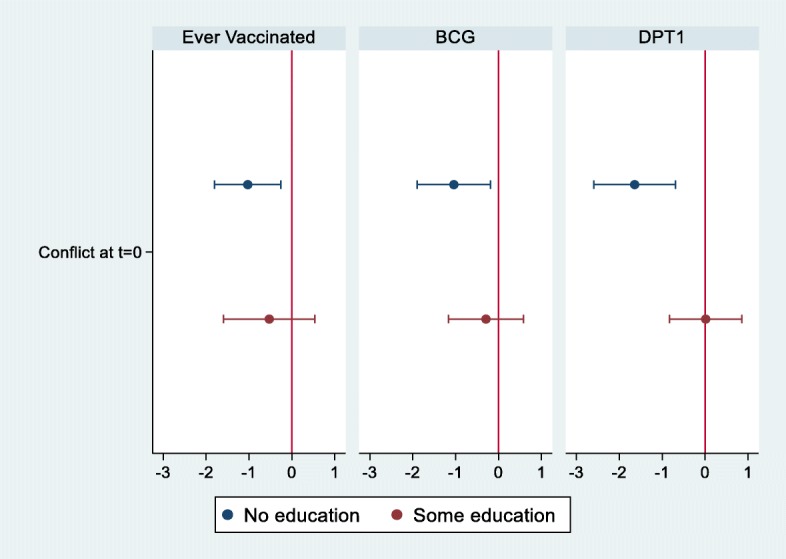
Effect of Conflict on Vaccination by Education Status. The sample is 2413 mothers who are in Borno, Yobe and Adamawa state. The figure shows the estimated coefficients, and the estimate is based on the logistic regression. The independent variable “conflict” takes 1 if the conflict occurred within 10 km from each mother. t = 0 means conflict at Birth month. Covariates include child’s age, birth year, sex of child, birth order, age of mother, education level of mother, marital status, religion, number of HH members, number of children under 5, wealth level

Figure 3 and Table 3 present the effect of conflict events on children’s vaccination according to the education level of the mothers. Conflicts have a differential effect according to the education level of the mothers (Fig. 3). For children of mothers without educational attainment, conflict at the time of birth decreases the odds of vaccination by 64.3% for any vaccines, by 64.6% for BCG, and by 81.6% for DPT1 (Table 3 Panel A). In contrast, for children of mothers with some educational attainment, conflict is not significantly associated with a reduced likelihood of vaccination, regardless of the type of vaccine (Table 3 Panel B).

**Table 3:** Effect of Conflict on Vaccination by Education Level (Odds Ratio)

	Ever vaccinated (1)	BCG (2)	DPT1 (3)
Panel A: No Education			
Conflict at Birth month	0.357*** [0.165,0.774]	0.354** [0.150,0.831]	0.194*** [0.075,0.500]
N	1629	1624	1620
	(1)	(2)	(3)
Panel B: Some Education			
Conflict at Birth month	0.590 [0.203,1.710]	0.747 [0.313,1.786]	1.013 [0.435,2.360]
N	674	749	735
Covariates	X	X	X

Notes: The sample is 2413 mothers who are in Borno, Yobe and Adamawa state

We use logistic regression. The independent variable “conflict” takes 1 if the conflict occurred within 10 km from each mother. Covariates include child’s age, birth year, sex of child, birth order, age of mother, education level of mother, marital status, religion, number of HH members, number of children under 5, wealth level

** significant at 5%, and *** significant at 1%

Table 4 presents the effect of conflict on the vaccination of children according to wealth level. For children in poor households, there is no significant association between the occurrence of conflict events and the likelihood of vaccination, although there is a large effect on the likelihood of ever getting vaccinated (Panel A). In contrast, among children in non-poor households, conflict at the time of birth decreases the odds of vaccination by 41.8% for any vaccines, by 49.2% for BCG, and by 51.9% for DPT1 (Panel A).

**Table 4:** Effect of conflict on vaccination by wealth level (odds ratio)

	Ever vaccinated (1)	BCG (2)	DPT1 (3)
Panel A: Poor			
Conflict at Birth month	0.261 [0.023,3.007]	1.000 [1.000,1.000]	0.400 [0.028,5.628]
N	1482	1467	1472
	(1)	(2)	(3)
Panel B: Non-Poor			
Conflict at Birth month	0.392*** [0.197,0.779]	0.508** [0.268,0.963]	0.491** [0.256,0.943]
N	919	919	899
Covariates	X	X	X

Notes: The sample is 2413 mothers who are in Borno, Yobe and Adamawa state

We use logistic regression. The independent variable “conflict” takes 1 if the conflict occurred within 10 km from each mother. “Poor” includes poorest and poorer, and “Non-poor” includes middle, richer, and richest. Covariates include child’s age, birth year, sex of child, birth order, age of mother, education level of mother, marital status, religion, number of HH members, number of children under 5, wealth level

** significant at 5%, and *** significant at 1%

## Discussion

Northeastern Nigeria has struggled with insecurity due to the Boko Haram insurgency over the past decade. Persistent armed conflicts can have disastrous effects on the population’s health. This study evaluates the effect of conflicts around the time of childbirth on the vaccination of children using detailed information about children’s birth months and the months that armed conflicts occurred.

Northeastern Nigeria has very low average vaccination rates compared to other parts of Nigeria. According to the National Immunization Coverage Survey conducted in 2016 and 2017, 42% of children in the northeast have never received any vaccination, while this is true of only 8 % in the southeast (National Immunization Coverage Survey, 2016/2017). The risk of disease increases in places with low vaccination rates [15]. Furthermore, conflict events can augment the risk of disease [6, 16].

The data clearly show that Boko Haram has purposely targeted urban neighborhoods where residents are more educated and wealthier. This finding is consistent with the existing literature [3].

We find that conflict events that occurred around the time of childbirths significantly decrease the likelihood that a child gets immunized. The effect of conflict events is large; if the conflict event occurred around the time of the childbirth, the likelihood of early immunization decreases by almost half. Conflict events that occurred around childbirth have a larger effect on the vaccination than do conflict events that occurred several months after birth. This differential effect of conflict occurrences according to timing implies that the effect of conflict on vaccination is time-sensitive. This is because the earliest scheduled vaccine is BCG at birth. If the conflict occurred only after several months, it is possible that a child has already received BCG and other vaccines that are due prior to the conflict events.

The differential effect of conflict events is worth noting. While the prevalence of conflict events is much higher among the educated population, the effect of conflict events on the vaccination of children of educated mothers is null. In contrast, conflict events have a strong negative effect on the vaccination of children of non-educated mothers, although the prevalence of conflict events is much lower among this non-educated population. This result implies that the effect of conflict events on health outcomes is severer among the non-educated population.

Because educational attainment is often correlated with wealth, we also examine the effect of conflict events according to wealth level. Counterintuitively, the negative effect of conflict events was significant only among the non-poor population. However, although insignificant, the effect size of conflict events is larger for “ever vaccinated” and DPT1 among the poor population than among the non-poor population. The insignificant result among the poor might be due to the very low prevalence of conflict events among the poor, as shown in Table 1.

Overall, armed conflicts had devastating effects on the vaccination of young children who were born at the time of these conflicts in northeastern Nigeria. Although the Boko Haram insurgency was concentrated in the wealthy neighborhoods of urban cities, the effect of the conflict was the most severe among the non-educated population.

### Limitations

There are several limitations to this study. First, the analysis does not identify the mechanisms through which conflict decreases the vaccination rate. It could be that the conflict destroys the health system and vaccination services were not available during the conflict period. It is also possible that the conflict events prevented people from accessing any services in the town due to insecurity. Second, due to the low prevalence of armed conflicts among the poor, a deep investigation of the effect of conflicts among them was infeasible. Third, this study does not evaluate the long-term effect of early exposure to armed conflicts on vaccination.

Future studies should address these limitations to deepen our knowledge of the effect of armed conflicts on vaccination behavior. Future studies should further explore 1) the mechanisms through which armed conflicts influence vaccine take-up from both the supply and demand sides, 2) the effect of armed conflicts on health behavior among the poor, and 3) the long-term consequences of armed conflicts on repeated vaccination behaviors such as dropout and timeliness of vaccinations.

## Conclusions

This paper evaluates the effect of armed conflicts in three northeastern states of Nigeria, Adamawa, Borno, and Yobe, that have been heavily affected by the Boko Haram insurgency for the past decade. We find that conflict events around the time of childbirth decrease the likelihood of any vaccination by almost half. We further find that this negative effect is significant among children of non-educated mothers, but not among children of educated mothers. The reduced vaccination rate puts the vulnerable population in the region at risk of contracting vaccine-preventable diseases. Future studies should further explore the mechanisms through which armed conflicts influence vaccine take-up, as well as their long-term consequences, especially among the poor.

## Data Availability

The datasets used during the current study are publicly available from https://ucdp.uu.se/ and https://dhsprogram.com/what-we-do/survey/survey-display-438.cfm.

## Appendix 1

**Table 5:** Descriptive statistics

	N (1)	% (2)	Mean (3)	Std. Dev (4)
Mother				
Age			28.66	7.16
Education				
No education	1639	67.9		
Primary	330	13.7		
Secondary	352	14.6		
Higher	92	3.8		
Religion				
Muslim	2129	88.2		
Non-muslim	284	11.8		
Marital Status				
Married	2318	96.1		
Not married	95	3.9		
Household				
Number of HH members			7.75	3.85
Number of children under 5			2.44	1.22
Wealth index				
Poorest	843	34.9		
Poorer	650	26.9		
Middle	412	17.1		
Richer	321	13.3		
Richest	187	7.7		
Urban/Rural				
Rural	1674	69.4		
Urban	739	30.6		
Children				
Age			29.19	17.44
Sex				
girl	1152	47.7		
boy	1261	52.3		
Birth order			4.17	2.77
Vaccination				
Ever vaccinated				
Yes	1184	49.1		
Never	1229	50.9		
BCG				
Yes	969	40.2		
No	1439	59.8		
DPT 1				
Yes	921	38.3		
No	1483	61.7		

Notes: The sample is 2413 mothers who are in Borno, Yobe and Adamawa state

## Appendix 2

**Table 6:** Effect of conflict on vaccination (odds ratio) (full model)

	Ever vaccinated (1)	BCG (2)	DPT1 (3)
Conflict at Birth month	0.528**[0.309,0.901]	0.550** [0.321,0.942]	0.520** [0.303,0.892]
Mother’s age	0.995 [0.971,1.020]	1.034*** [1.008,1.060]	1.020 [0.994,1.046]
Mother’s education (Comparison: No education)			
Primary	2.661*** [2.000,3.542]	2.651*** [1.996,3.521]	3.156*** [2.365,4.211]
Secondary	6.097*** [4.185,8.884]	5.424*** [3.834,7.672]	5.063*** [3.600,7.121]
Higher	3.840*** [1.914,7.702]	4.255*** [2.169,8.346]	5.855*** [2.969,11.546]
Muslim	0.407*** [0.281,0.590]	0.278*** [0.193,0.401]	0.293*** [0.202,0.424]
Married	0.384*** [0.209,0.704]	0.501** [0.284,0.882]	0.452*** [0.255,0.803]
Number of HH members	1.055*** [1.014,1.098]	1.041** [1.000,1.084]	1.045** [1.003,1.089]
Number of children under 5	0.868** [0.778,0.970]	0.935 [0.835,1.048]	0.933 [0.831,1.046]
Wealth index (Comparison = poorest)			
Poorer	1.844*** [1.439,2.362]	2.022*** [1.548,2.641]	2.572*** [1.954,3.385]
Middle	2.695*** [1.982,3.664]	2.599*** [1.880,3.593]	3.324*** [2.384,4.635]
Richer	5.128*** [3.425,7.678]	5.753*** [3.832,8.637]	6.260*** [4.135,9.478]
Richest	6.224*** [3.504,11.055]	8.914*** [5.062,15.698]	8.964*** [5.144,15.621]
Rural	1.143 [0.864,1.513]	1.154 [0.866,1.539]	1.493*** [1.113,2.003]
Child = girl	1.109 [0.912,1.347]	1.072 [0.874,1.314]	0.981 [0.797,1.207]
Birth order	1.063* [0.997,1.132]	0.997 [0.934,1.064]	1.034 [0.968,1.105]
N	2410	2405	2401

Notes: The sample is 2413 mothers who are in Borno, Yobe and Adamawa state. We use logistic regression. The main independent variable “conflict” takes 1 if the conflict occurred within 30 km from each mother. Covariates include child’s age and birth year&month

** significant at 5%, and *** significant at 1%

## Appendix 3

**Table 7:** Effect of conflict on vaccination (30 km)

	Ever vaccinated (1)	BCG (2)	DPT1 (3)	Ever vaccinated (4)	BCG (5)	DPT1 (6)	Ever vaccinated (7)	BCG (8)	DPT1 (9)	Ever vaccinated (10)	BCG (11)	DPT1 (12)
Conflict at Birth month	0.537*** [0.339,0.850]	0.555** [0.347,0.888]	0.530*** [0.329,0.854]									
Conflict at 1 month after birth				0.607** [0.386,0.954]	0.445*** [0.278,0.713]	0.569** [0.355,0.914]						
Conflict at 2 month after birth							0.609** [0.396,0.936]	0.539*** [0.345,0.843]	0.487*** [0.306,0.774]			
Conflict at 3 month after birth										0.682* [0.455,1.022]	0.622** [0.410,0.944]	0.776 [0.507,1.187]
N	2410	2405	2401	2410	2405	2401	2410	2405	2401	2410	2405	2401
Covariates	X	X	X	X	X	X	X	X	X	X	X	X

Notes: The sample is 2413 mothers who are in Borno, Yobe and Adamawa state. We use logistic regression. The independent variable “conflict” takes 1 if the conflict occurred within 30 km from each mother. Covariates include child’s age, birth year, sex of child, birth order, age of mother, education level of mother, marital status, religion, number of HH members, number of children under 5, wealth level

* significant at 10%, ** significant at 5%, and *** significant at 1%
